# Relationship of environmental factors in pond water and dynamic changes of gut microbes of sea bass *Lateolabrax japonicus*

**DOI:** 10.3389/fmicb.2023.1086471

**Published:** 2023-03-30

**Authors:** Zheng Zhu, Yu-Min Xu, Jun-Han Liang, Wei Huang, Jin-Ding Chen, Si-Ting Wu, Xiao-Hong Huang, You-Hua Huang, Xiao-Yang Zhang, Hong-Yan Sun, Qi-Wei Qin

**Affiliations:** ^1^Guangdong Laboratory for Lingnan Modern Agriculture, College of Marine Sciences, South China Agricultural University, Guangzhou, Guangdong, China; ^2^College of Veterinary Medicine, South China Agricultural University, Guangzhou, Guangdong, China; ^3^Southern Marine Science and Engineering Guangdong Laboratory, Zhuhai Yueshun Aquaculture Co., Ltd., Zhuhai, China; ^4^Laboratory for Marine Biology and Biotechnology, Qingdao, China; ^5^Qingdao National Laboratory for Marine Science and Technology, Qingdao, China

**Keywords:** gut microbes, bacterial community, environmental factors, *Lateolabrax japonicus*, sea bass ponds

## Abstract

The effect of structure of gut microbes on the health of host has attracted increasing attention. Sea bass *Lateolabrax japonicus* is an important farmed fish in China. The relationship of the dynamic changes of intestinal bacterial communities in *L. japonicus* and the cultural water environment is very important for healthy culture. Here, the diversity and abundance of the gut microbial communities of *L. japonicus* were evaluated during the culture using 16S rRNA Illumina sequencing. Both the opportunistic pathogens *Aeromonas* (1.68%), *Vibrio* (1.59%), and *Acinetobacter* (1.22%); and the potential probiotics *Lactobacillus* (2.27%), *Bacillus* (1.16%), and *Lactococcus* (0.37%) were distributed in the gut of *L. japonicus*. The increasing concentration of nitrogen of water environments with the increase of culture time significantly correlated with shifts in the microbial community structure: 40.04% of gut microbial changes due to nitrogen concentration. Higher concentrations of nitrogen showed a significantly negative correlation with intestinal probiotics in *L. japonicus*. The results indicate that the abundance of intestinal bacteria of *L. japonicus* is mainly driven by the changes of environmental factors (e.g., nitrogen), and it’s very important that the linking environmental parameters with bacterial data of guts could be used as an early warning indicator in *L. japonicus* heath culture.

## Introduction

Gut microorganisms play a crucial role in the growth, development and health of the host, and the guts of fish also have a large number of bacteria (Jandhyala et al., 2015; Diwan et al., 2022). These bacterial communities are consist of probiotics, opportunistic bacteria, and neutral bacteria (Lv et al., 2021). Probiotics, one of important components of intestinal microorganisms, can exert immunoregulatory function, inhibit the proliferation of intestinal pathogens, and protect the integrity of the intestinal mucosal barrier (Cristofori et al., 2021). The gut is also a reservoir of pathogens. Normally, enteric pathogens are at a low abundance under interaction with intestinal probiotics (Vargas-Albores et al., 2021). However, the abundances of beneficial bacteria and pathogenic bacteria were different in diseased fish (Xiao et al., 2021; Yang et al., 2022), indicating that gut microbes might be closely related to the health of the host. It’s useful for controlling the diseases outbreak of fish according to the composition of the gut microbiota (Xiong et al., 2019; Das et al., 2022; Medina-Félix et al., 2022).

Fish live in the complex water environments, and the relationship between its gut microbes and the water environment is crucial for healthful culture (Jing et al., 2021). Deterioration of the water environment can cause various diseases of aquatic animals (Lieke et al., 2020). Dynamic changes of gut microbes of fish are influenced by both the fish themselves and environmental factors of water (Chen et al., 2022). Understanding the impact of the water environment on gut microbes is important to prevent aquaculture diseases, increase yields, and improve food quality (Wei et al., 2021; Chen et al., 2022).

Sea bass *Lateolabrax japonicus* is an important farmed fish in China. In 2021, the production of the farmed *L. japonicus* in China exceeded 199,000 tons (Fisheries Bureau of the Ministry of Agriculture, 2022). South China Sea is the major production areas, and the annual production of sea bass accounts for more than half of China (Zhao et al., 2018b; Zhang et al., 2022). At present, pond culture is the main culture method of *L. japonicus* in China. However, the water environment of the pond culture is relatively independent and has poor self-purification ability (Meerhoff and de los Ángeles González-Sagrario, 2022). Undigested feed, excreta and biological carcasses increase the concentration of nitrogen and phosphorus of water, and exceed the self-purification ability of water during culture (Liu et al., 2021). Bad water environment would directly lead to slow growth, yield reduction, disease outbreak, and even death of cultured fish (Boyd, 2017; Das et al., 2022).

Basis on this information, the structure of gut microbes of the farmed *L. japonicus*, and the relationship between gut microbes and the environmental factors of water were analyzed in this study, which would provide a theoretical basis for healthy culture of *L. japonicus*.

## Materials and methods

### Sample collection

The experimental pool was located in Zhuhai, China (113^°^14’57”E, 22^°^7’39.71”N), with an area of about 9,720 square meters (135 m × 72 m) and an average water depth of 2 m. The experimental sea bass was fed twice a day at a ratio of 3% body weight with puffed formula feed (Guangdong Yuehai Feed Group Co., Ltd., Zhanjiang, China).

The breeding cycle of sea bass in Guangdong, China is 1 year, and the environmental factors and climates were different in the four seasons. The times of sampling were set in the four seasons (January, April, August, and October). For each sampling, 1.8 m of water below the water surface was collected using a 2.5 L plexiglas water collector, filtered using 0.45 μM filtering membrane, and then used for analysis. Sixteen *L. japonicus* were randomly collected from the pond, and the length and the weight per fish were measurements. Contents from the second half of the gut of six *L. japonicus* were collected, and immediately frozen in liquid nitrogen for further analysis.

### Determination of water physicochemical factors

Temperature and pH of the water were measured using a pH-meter (Sartorius, Göttingen, Germany). Total phosphorus, phosphate, total nitrogen, nitrate, nitrite, and ammonium nitrogen of cultured water were determined according to standard methods using UV-VIS spectrophotometer (P general, Beijing, China) (Zou and Cheng, 2002).

### DNA extraction, PCR and sequencing

Microbial DNA of 100 mg of intestinal contents was extracted using the DNeasy Power Soil Pro Kit (QIANEN, Germany) according to the manufacturer’s instructions. DNA integrity was assessed using 2% agarose gel electrophoresis. DNA purity and concentration were tested using NanoDrop2000 (Thermo Fisher, USA). A total of 10 ng of genomic DNA was added to the polymerase chain reaction (PCR) system. PCR was performed to amplify the V4 region of the 16S rDNA gene using primers 515F (5’-GTGCCAGCMGCCGCGG-3’) and 806R (5’-GGACTACHVGGGTWTCTAAT-3’) with barcode. Each sample was performed in triplicates. PCR products were detected by 2% agarose gel electrophoresis, recovered using the AxyPrep DNA Gel Recovery Kit (AXYGEN, China), and quantified with QuantiFluor™ –ST blue fluorescence quantification system (Promega, USA). High-throughput sequencing was performed on the Illumina MiSeq platform (Illumina, USA) according to standard protocols developed by Majorbio Bio-Pharm Technology Co, Ltd (Shanghai, China).

### Bioinformatic analysis

The raw data obtained were stitched into sequences, and the sequences were filtered. A total of 97% similarity sequences were sorted into the same OTU using Uparse 7.0.1090 software. The representative sequences of each OTU were aligned according to the silva database^1^ and classified using RDP Classifier. Principal co-ordinates analysis (PCoA), analysis of similarities (ANOSIM), Canonical correspondence analysis (CCA), variance partitioning analysis (VPA) and mantel test analysis were performed by the vegan 2.4-2 package of R 4.1.2 software. Bacterial taxonomic tree was carried out by Mega X. Species abundance heatmaps were carried out with the gplots 2.17.0 package of R 4.1.2. Collinear relation diagram is carried out by Circos 0.69-8 software. Bacterial FAPROTAX Function was predicted by FAPROTAX 1.2.6 software. Bacterial PICRUSt2 Function was predicted by PICRUSt2 2.2.0 software. Network analysis was carried out by QIIME 1.9.1 software. Diversity indices and bacterial abundances of different groups were compared using one-way ANOVA. Statistical significance was set at *P* < 0.05.

## Results

### Growth of *L. japonicus* and water physicochemical factors

**FIGURE 1:**
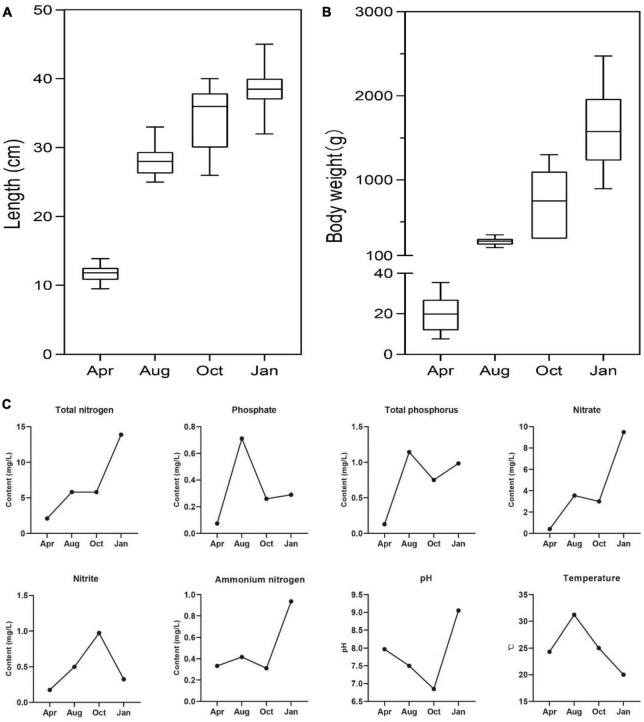
The growth status of farmed sea bass and the physicochemical factors of water. **(A)** The changes of sea bass body length. **(B)** The changes of sea bass body weight. **(C)** The changes of total phosphorus, phosphate, total nitrogen, nitrate, nitrite, ammonium nitrogen, pH, temperature in water.

Culture cycle of *L. japonicus* in South China was about 1 year, so the growth of *L. japonicus* and the changes of environmental factors in the four seasons were explored. As expected, the body length and body weight of *L. japonicus* were increased gradually with the time of culture (Figures 1A, B). Higher temperatures of water occurred in August, and lower water temperatures occurred in January (Figure 1C). There was little difference of water temperature in April and October. This result was consistent with the climatic conditions in the experimental locality. In the early stage of culture (April), the concentration of both nitrogen and phosphorus of cultured water were at a low level. With the increase of culture time, the concentration of total nitrogen, nitrate and ammonium nitrogen showed an increasing trend. The concentration of total phosphorus and phosphate was the highest in August. The highest concentration of nitrite was in October.

### Diversity of gut microbes

A total of 2,642,153 sequences were obtained from 24 samples, with an average length of 256 bp. A total of 2,609,399 of these sequences are high-quality valid sequences, with an average coverage rate of 98.76%. Shannon, Simpson, Chao and Sobs rarefaction curves also indicate that all sample sequences have sufficient coverage.

**FIGURE 2:**
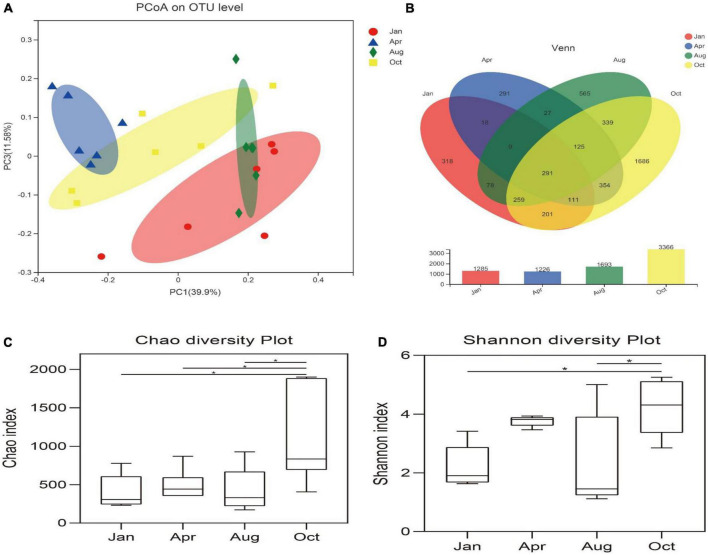
Diversity of gut microbes. **(A)** Principal co-ordinates analysis (PCoA) was performed at the OUT level based on weighted UniFrac distances for all samples. Different colored dots in the figure represent different groups. The x-axis and y-axis (PCoA1 and PCoA3) represent 33.9 and 11.58% of the variance, respectively. **(B)** Venn diagram represents sharing and unique OUT in Jan, Apr, Aug, Oct. Histograms represent the total number of OUT for each group. **(C)** Chao index indicates community distribution abundance. **(D)** Shannon index indicates diversity in community distribution. *p < 0.05.

In order to analyze the diversity and abundance of gut microbial communities of *L. japonicus* at differential cultured times, PCoA was performed using weighted UniFrac distances. As shown in Figure 2A, There was no significant difference of the microbial community between January and August (ANOSIM, *P* > 0.05), but significant difference occurred in the remaining time points (ANOSIM, *P* < 0.05).

The alpha diversity of the gut microbes was conducted using the Chao index, Shannon index and the number of OTUs. Based on 97% homology, high-quality valid sequences can be divided into 4,672 OUTs (Figure 2B). The Chao index of the gut microbes in October was significantly higher than that of other groups (*P* < 0.05), and there was no significant difference in the Chao index between other time points (*P* > 0.05) (Figure 2C). The Shannon index of the gut microbes in October was significantly higher than that in January and August (*P* < 0.05) (Figure 2D).

### Composition and changes of gut microbes in *L. japonicus*

**FIGURE 3:**
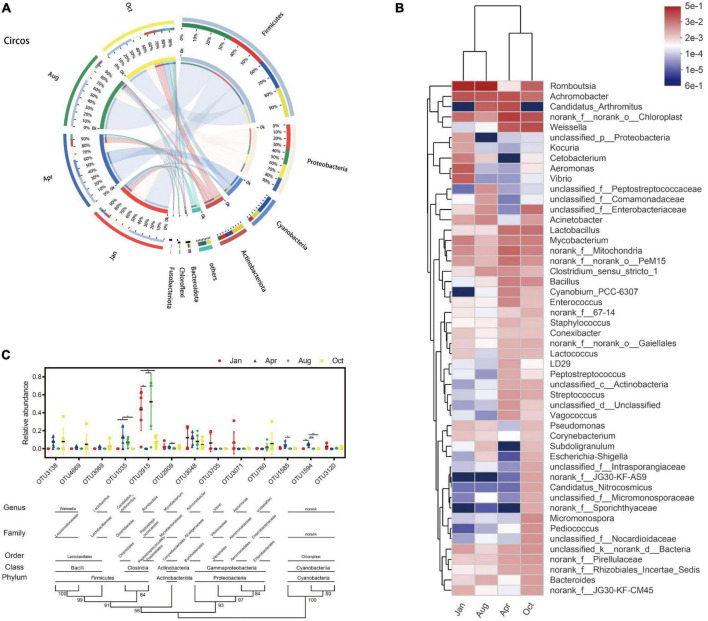
Composition and dynamic changes of gut microbes. **(A)** Collinear relation diagram between bacteria phylum and each group. Only phyla with an average abundance greater than 1% are presented, and the rest are merged into others. **(B)** Heatmap of bacterial genus level in each group. Only the top 50 abundance bacterial genera are shown in the figure. Bacterial genera and groups were clustered using the average algorithm. The color of the color blocks was used to indicate the relative abundance of each genus. **(C)** Classification and change of main OUT in each group. Only OUT with mean abundances greater than 1% are shown in the figure. OUT presented in the figure is clustered using the N-J algorithm. The values of the cluster tree represent the degree of reliability of this branch. Above the cluster tree are species classifications corresponding to OUT. Scatter plots were used to represent the abundance of OUT and mean ± standard error were presented. *p < 0.05, ^**^p < 0.01.

All 4,672 OUTs can be divided into 58 phylum, 160 classes, 356 orders, 612 family, and 1,318 genus. At the phylum classification level, the gut microbes of *L. japonicus* were mainly composed of *Firmicutes* (48.70%), *Proteobacteria* (22.65%), *Cyanobacteria* (10.75%), *Actinobacteriota* (9.71%), *Bacteroidota* (1.65%), and *Planctomycetota* (1.27%) (Figure 3A). Compared with that of April, the abundance of *Cyanobacteria* (*P* < 0.01), *Actinobacteriota* (*P* < 0.05) and *Planctomycetota* (*P* < 0.05) in October was significantly decreased. There were no significant differences in other major bacterial phyla between groups. As for genus, the predominant bacteria were *Romboutsia* (25.85%), *Achromobacter* (8.95%), *Weissella* (5.47%), *Candidatus Arthromitus* (4.92%), *Mycobacterium* (2.33%), *Lactobacillus* (2.27%), *Aeromonas* (1.68%), *Vibrio* (1.59%), *Acinetobacter* (1.22%), and *Bacillus* (1.16%) (Figure 3B). At the OUT level, the gut microbes were mainly from 13 OUTs. As shown in Figure 3C, OTU1035 (*Candidatus Arthromitus*), OUT1594 (*Chloroplast*) were undetectable in January. OTU1035 was significantly enriched in April and August. OTU2915 (*Romboutsia*) was significantly enriched in January and August. OTU1585 (*Chloroplast*) and OTU1594 were only significantly enriched in April.

### The relationship between environmental factors and gut microbes

**FIGURE 4:**
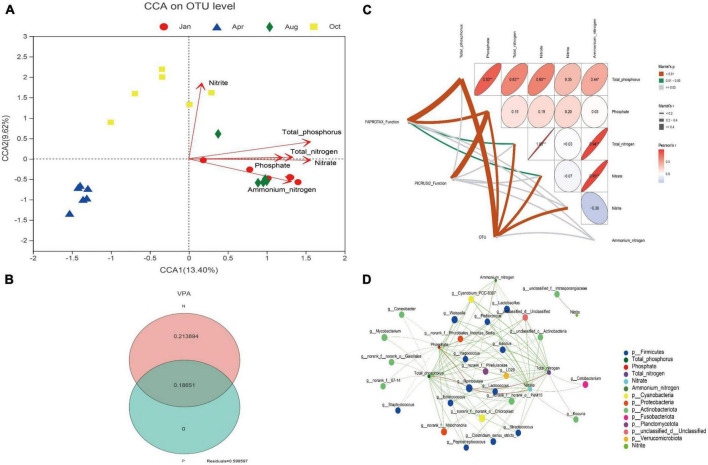
Relationship between nitrogen and phosphorus with gut microbes. **(A)** Canonical correspondence analysis (CCA) plot for OUT level. Different colored dots in the figure represent different groups. Red arrows are corresponding environmental factors. The length of arrows can represent the relative influence of environmental factors on OTU data. **(B)** Variance partitioning analysis (VPA) venn diagram. N indicates the explanation degree of changes in gut microbiota composition by total nitrogen, nitrate, nitrite and ammonium nitrogen in water. P indicates the explanation degree of changes in gut microbiota composition by total phosphorus and phosphate. **(C)** Mantel test analysis plot. Matrices represent correlations between environmental factors. In the matrix, the direction of the ellipse represents positive or negative correlation. The color and value of the ellipse represent the pearson correlation coefficient. *p < 0.05, **p < 0.01. The width of the line between FAPROTAX Function, PICRUSt2 function, and OUT to the environmental factor in the matrix represents the magnitude of their pearson correlation coefficient. The color of the line represents the significance of the correlation. **(D)** Network analysis plot. Pearson correlation coefficients were calculated between the top 50 bacterial genera in mean abundance and environmental factors. Nodes with absolute correlation coefficients greater than 0.5 and *p* < 0.05 were plotted. The circles in the figure represent different environmental agents or bacterial genera. The size of the circle represents the magnitude of the bacterial genus abundance or environmental factor values. Different colors represent different bacterial phyla or environmental agents. The color of the line indicates positive or negative correlation. Red indicates positive correlation, and green indicates negative correlation. The width of the line indicates the magnitude of the correlation coefficient.

The effect of environmental factors of culture water on gut microbes was conducted by CCA. As shown in Figure 4A, The concentrations of total phosphorus, phosphate, total nitrogen, nitrate, nitrite or ammonium nitrogen had significant effects on gut microbes in *L. japonicus* (*P* < 0.01). VPA was used to further quantify the effect of the concentration of nitrogen and phosphorus on the gut microbes. The results showed that the concentration of nitrogen and phosphorus could explain 40.04% of the changes of gut microbes: 18.65% were explained by both nitrogen and phosphorus, 21.39% by nitrogen alone, and 0% by phosphorus alone (Figure 4B). Analysis of mantel test was used to reveal the correlation between the environment factors and the gut microbial function. As shown in Figure 4C, The FAPROTAX function of gut microbes had a significant correlation with the environment factors total phosphorus, phosphate, total nitrogen and nitrate (*P* < 0.01 or *P* < 0.05); the PICRUSt2 function of gut microbes was only significantly correlated with the phosphate (*P* < 0.01); the OUT of gut microbes was significantly correlated with total phosphorus, phosphate, total nitrogen, nitrate and nitrite (*P* < 0.01).

To further search for bacterial genera associated with environmental factors, network analysis was performed. As shown in Figure 4D, Only one unclassified genus *Intrasporangiaceae* was significantly correlated with the concentration of nitrite (*P* < 0.05); *Romboutsia* was the only genus hat had a significant positive correlation with total phosphorus, phosphate, total nitrogen, and nitrate of water (*P* < 0.05); *Kocuria* and *Cetobacterium* had significant positive correlation with total nitrogen and nitrate (*P* < 0.05); and the other intestinal bacteria genera, especially *Lactobacillus*, *Bacillus*, and *Lactococcus*, were significantly negatively correlated with nitrogen and phosphorus (*P* < 0.05). The further analysis found that, the abundance of *Lactobacillus*, *Bacillus* and *Lactococcus* decreased by 96% (July), 98% (October), and 41% (January), respectively, compared with that of April (the beginning of the culture).

## Discussion

Gut microbes play an important role in the biological processing and the health of the hosts. In this study, the diversity, dynamic changes of gut bacteria and the environmental factors of water were explored during the *L. japonicus* culture.

The intestines of fish are important repositories of bacteria (Zhao et al., 2018a). Understanding the species of intestinal bacteria (e.g., pathogenic bacteria) of fish are important for its healthy culture (Tarnecki et al., 2017; Ou et al., 2021). This study showed that *Aeromonas* (1.68%), *Vibrio* (1.59%), and *Acinetobacter* (1.22%) are major members of gut microbes in farmed *L. japonicus*. The presence of the opportunistic pathogens is a non-negligible threat to *L. japonicus*. *Aeromonas* is widely distributed in aquatic ecosystems, and the gut of various aquatic animals (Pessoa et al., 2019). Some *Aeromonas* (e.g., *A. hydrophila*) have been proven to be significant pathogen to fish, and cause fish disease when fish was a low immunity or stressful conditions (Nhinh et al., 2021). *Vibrio* is one of the most important pathogens of fish, and causes huge economic losses to the fish industry every year (Sheikh et al., 2022). Members of the *Vibrio* have a strong ability to generate biofilms, and therefore *Vibrio* possess strong tolerance to antimicrobials and the host immune system (Arunkumar et al., 2020). Previous studies have shown that *Acinetobacter* only causes the infection of humans (Carvalheira et al., 2021). But now, some members of the *Acinetobacter* genus can cause the diseases of fish (Cao et al., 2016; Kuebutornye et al., 2020). Potential pathogens in the gut microbiota can cause damage when host immunity is low (Pérez-Sánchez et al., 2018).

Microorganisms in the surrounding environment are one of the important sources of gut microbes in fish (Sun et al., 2019). *Aeromonas*, *Vibrio*, and *Acinetobacter* are common residents in water environment (Diwan et al., 2022). Our study showed that these opportunistic pathogens are also able to colonize the intestinal tract of *L. japonicus*. Normally, the intestinal mucosal immunity and mucosal barrier of fish can control the composition of intestinal microorganisms and prevent the massive proliferation and invasion of fish pathogens (Okumura and Takeda, 2018). However, when the environment changes dramatically or outbreaks occur during aquaculture, these pathogenic bacteria will proliferate abnormally and further aggravate the condition. In the past, multiple antibiotics were extensively used response to bacterial infections during culture. However, the abuse of antibiotics leads to serious food safety, environmental pollution, and bacterial resistance problems. Therefore, it is particularly important to use a more environmentally friendly and safe method to shape the gut microbes of *L. japonicus* to reduce bacterial diseases during *L. japonicus* culture (Shao et al., 2021).

Probiotics are widely used for enhancing immunity, helping digestion, improving water quality, and promoting growth and reproduction (Banerjee and Ray, 2017; Yilmaz et al., 2022). In this study, the potential probiotics *Lactobacillus* (2.27%), *Bacillus* (1.16%), and *Lactococcus* (0.37%) were detected from the intestines of *L. japonicus*, indicating that these bacteria have good colonization ability, and can be applied in its culture. *Lactobacillus* and *Lactococcus* are both lactic acid-producing bacteria and are important for enhancing immunity and disease resistance in animals (Van Doan et al., 2020). *Bacillus* as a probiotic has been shown to improve feed utilization thereby promoting growth and enhancing immunity to combat disease, while improving aquaculture water quality (Kuebutornye et al., 2019; Hlordzi et al., 2020).

With the increase of farming time in *L. japonicus* pond, the concentration of nitrogen and phosphorus of the water also increased continuously. In the middle and late stage of culture, the food intake of *L. japonicus* increased rapidly, and the faces at the bottom continued to accumulate, resulting the enrichment of nitrogen and phosphorus in the water. Previous studies have shown that fish only absorb about 20% of the phosphorus in the feed, and majority of phosphorus in the feed was released into the water (Hua and Bureau, 2006; Kong et al., 2020). High concentration of phosphorus of water can cause the overgrowth of algae and weeds, as well as oxygen depletion and toxin production (Kong et al., 2020). The enrichment of ammonia nitrogen of water might change the pH of fish blood and reduce the ability of blood to carry oxygen (Xu et al., 2021). High concentration of ammonia nitrogen of culture water will lead to oxidative stress of fish, resulting in a decrease of antioxidant enzymes and the activity of superoxide dismutase (Xu et al., 2021). Meanwhile, ammonia stress can directly damage the gut of fish and cause immuno-suppression (Xu et al., 2021). The gut mucosal immunity of fish is an important means to control the composition of gut microbes (Brown et al., 2013; Yoo et al., 2020). When fish were cultured in a high concentration of nitrogen-phosphorus, the composition and function of the gut microbiota would be affected (Li et al., 2021). Analysis of VPA here showed that nitrogen of water was able to explain 40.04% of the changes of gut microbes, and 18.65% of the changes the gut microbes was explained by both nitrogen and phosphorus of water. The gut microbiota has multiple metabolic and biosynthetic functions, so it is more important to study the function of gut microbes (Torsvik and Øvreås, 2002; Bosco and Noti, 2021). Mantel test analysis showed a significant correlation between phosphate and the PICRUSt2 function of the gut microbiota (*P* < 0.01). FAPROTAX mainly predicts the metabolic function of gut microbes to substances such as nitrogen and phosphorus in the environment. FAPROTAX function was significantly correlated with total phosphorus, phosphate, total nitrogen and nitrate (*P* < 0.01 or *P* < 0.05). Therefore, the function of gut microbes of *L. japonicus* can be changed by regulating environmental factors of pond.

Network analysis showed that the concentrations of nitrogen and phosphorus of water were significantly negatively correlated with most bacterial genera including the probiotics *Lactobacillus*, *Bacillus*, and *Lactococcus*. With the increasing of nitrogen and phosphorus during culture, the abundance of potential probiotics decreased. Therefore, the enrichment of nitrogen and phosphorus in the aqueous environment is not conducive to the colonization of intestinal probiotics. Feeding probiotics in *L. japonicus* pond culture should ensure that nitrogen and phosphorus in water are at a low level. *Romboutsia* can be isolated in the gut of a variety of animals both aquatic and terrestrial (Gerritsen et al., 2019). It has been shown that *Romboutsia* are significantly enriched in fish fed fishmeal (Habte-Tsion et al., 2022). *Romboutsia* was the only genus which showed a significant positive correlation with nitrogen and phosphorus in this study (*P* < *0.05*). *Romboutsia* may therefore be a manifestation of *L. japonicus* adaptation to nitrogen-phosphorus enriched environments. Studying the interaction between *Romboutsia* and the host is important for exploring the effects of nitrogen and phosphorus on the host.

## Conclusion

The diversity and abundance of gut bacterial communities of *L. japonicus* were studied, and its dynamic changes response to the environmental factors of water were elucidated. Both the opportunistic pathogens (*Aeromonas*, *Vibrio*, and *Acinetobacter*); and the potential probiotics (*Lactobacillus*, *Bacillus*, and *Lactococcus*) were distributed in the gut of *L. japonicus*. The environmental factors of water affect the diversity of bacterial communities. The concentration of nitrogen and phosphorus significantly drive the composition and function of gut microbes during culture, and higher concentration of nitrogen adversely could reduce the ratio of probiotics of gut bacterial communities in *L. japonicus*. The results would help us explore the relationship of environmental parameters of water and bacterial communities of guts, and would be used as an early warning indicator in *L. japonicus* heath culture.

## Data availability statement

The data presented in this study are deposited in the NCBI, accession number: PRJNA898861.

## Ethics statement

This animal study was reviewed and approved by the Ethics Committee of South China Agricultural University.

## Author contributions

Q-WQ, H-YS, and J-DC conceived the research. ZZ, Y-MX, J-HL, WH, and S-TW performed the experiments. ZZ wrote the manuscript. X-HH and Y-HH edited the manuscript. X-YZ contributed sampling or data analysis pipelines. All authors reviewed and approved the manuscript.

## Footnotes

1
https://www.arb-silva.de/
